# Datasets on probability distributions of arrival and departure times of privately used electric vehicles

**DOI:** 10.1016/j.dib.2024.110917

**Published:** 2024-09-07

**Authors:** Yan Wu, Syed Mahfuzul Aziz, Mohammed H. Haque

**Affiliations:** UniSA STEM, University of South Australia, Mawson Lakes, SA, Australia

**Keywords:** Electric vehicle (EV), Vehicle travel data, Probability distribution, Electric vehicle charging demand, Vehicle-to-home discharging, Energy cost optimisation

## Abstract

This article presents the data used in the paper “Vehicle-to-home operation and multi-location charging of electric vehicles for energy cost optimisation of households with photovoltaic system and battery energy storage” [1]. The datasets reported in this paper include the probability distributions of arrival and departure times of privately used vehicles at both home and workplace. The datasets relate to two types of privately used vehicles, namely, those commuting to and from the workplace, and those used for all other activities except attending the workplace. These two vehicle types are referred to as *work vehicles* and *casual vehicles* respectively. The datasets consider different daily travel frequencies, where the term *travel frequency* refers to the number of times a vehicle arrives at home each day. The raw data of vehicle usage is sourced from the Victorian Integrated Survey of Travel & Activity (VISTA) [2], which is an ongoing survey that has collected data from 32,000 households and 82,000 people since 2012. This dataset is filtered to obtain the arrival–departure times of privately-used *work vehicles*. For the *casual vehicles*, the filtered data is categorised based on daily travel frequency to obtain the arrival–departure times. Using the filtered and categorised data, the probability distributions of the arrival–departure times for *work* and *casual vehicles* are extracted. Microsoft Excel and MATLAB software are used to perform the required processing. The experimental methods used to obtain the required data, from downloading the raw datasets to extracting the probability distributions, are described in this paper.

Specifications Table

**Table:** 

Subject	Electrical Engineering
Specific subject area	Probability distribution of vehicle travel pattern, electric vehicle (EV) charging demand, energy cost optimisation
Data format	Raw, Filtered and categorised, Processed
Type of data	Excel files, Tables, Figures
Data collection	Raw travel dataset “VISTA by Trip 2012–2018.zip” is downloaded from Victorian Integrated Survey of Travel & Activity [2].
Data source location	Victorian Integrated Survey of Travel & Activity [2] Institution: Victoria State Government – Department of Transport City/Town/Region: Victoria Country: Australia
Data accessibility	Repository name: Mendeley Data Data identification number: 10.17632/gphwn7sy5n.1 Direct URL to data: https://data.mendeley.com/datasets/gphwn7sy5n/1
Related research article	Y. Wu, S. M. Aziz and M. H. Haque, Vehicle-to-home operation and multi-location charging of electric vehicles for energy cost optimisation of households with photovoltaic system and battery energy storage, Renewable Energy. 221 (2024), p. 119,729. https://doi.org/10.1016/j.renene.2023.119729. [1]

## 1 Value of the Data

• For any number of electric vehicles (EVs), the probability distributions of home/workplace *arrival* and *departure times* presented in this paper are useful to individually create the *arrival* and *departure times* of EVs with varying usage types.
• The created *arrival* and *departure times* can be used to generate EV charging demand profile for individual households based on the daily travel distance of the EVs. The household EV charging demand profile is useful to estimate the home energy costs due to charging. This can then be used to reduce the energy costs by developing electricity tariff-sensitive charging strategies based on the EV charging demand profile.
• The created *arrival* and *departure times* can also be used to generate the EV charging demand profile for the EV fleet of a workplace or a group of residences in an area. Such demand profiles for EV fleets are essential to analyse the impact of EV charging on the distribution networks [3].
• The above-mentioned charging demand profiles can be used for modelling and analysing the charging profiles for various EV usage types, which can assist with the planning and operation of distribution networks.
• Researchers, policymakers, potential EV buyers and distribution network operators can utilize the presented probability distributions of workplace/home *arrival–departure times* for planning and design purposes [4,5].
• Researchers can use the presented datasets to reproduce the results reported in the original research article [1], compare with the results based on other datasets from different regions/countries, and thereby contribute to advancing the state-of-the-art in sustainable EV integration research.

The original research article [1] used daily travel distances, as well as arrival and departure times at both home and work, to determine the energy demand of EVs at the respective charging locations. The method for obtaining the daily travel distances of privately used vehicles was presented in [6], which was used in a research article to determine the charging demand of a campus EV fleet [7]. This paper presents datasets of *arrival* and *departure times* at home and workplace.

## 2 Background

The electrification of transportation and the utilization of renewable energy sources are essential to reduce greenhouse gas emissions and to achieve sustainable development goals [8]. However, the expected increase in EV charging demand and the continuous growth of renewable generation are presenting new challenges to the existing power grid, for example, the “duck curve phenomenon” [3,9,10]. The latter represents the very low power demand on the grid during the daytime when solar electricity generation is high, and high power demand on the grid during mornings and evenings when solar generation is either very low or nil. Vehicle-to-home (V2H) operation and multi-location charging of EVs are sensible approaches to mitigate some of these challenges while helping individual customers reduce their household energy costs. V2H operation utilizes the electric vehicles as mobile batteries, supplying electricity to the house when needed, for example, during the evening peak periods when electricity prices are high for the majority of today's cost-reflective tariffs [11,12]. Multi-location charging can reduce the reliance of EVs on home charging, shifting the demand to workplace or public charging during the daytime periods when there is high solar generation. This would help utilize green energy for EV charging during the day when exporting the excess solar photovoltaic generation to the grid is not often an option due to very low power demand on the grid. Since the amount of charging required and the amount of discharging possible by EVs depend on the daily travel needs of the EVs, credible datasets are needed on the distance travelled, arrival and departure times so that the charging and discharging options can be determined. For a large fleet of EVs, it is necessary to determine the probability distributions of the vehicle arrival and departure times at/from the charging locations. The probability distributions help estimate the arrival and departure times of individual vehicles in a fleet. Based on the motivation presented above, this paper presents datasets on the probability distributions of EV *arrival* and *departure times* based on the processing of the raw datasets sourced from the VISTA website.

## 3 Data Description

The datasets reported in this paper are summarised in Table 1. These include one raw travel dataset, three filtered and categorised datasets, and three processed datasets.

**Table 1:** List of datasets available with this paper.

No.	Name	Type	Description
1	*T_VISTA1218_V1.csv* (including: *VISTA - Glossary of Variables 12–18.docx*)	Raw dataset	Contains travel distance, *arrival & departure times* of all trips during the day for all types of commuting modes (e.g., walking, bus, private vehicle and train).
2	*VISTA_Time_HomeDeAr_WorkVehicle.csv*	Filtered and categorised dataset	Home *departure & arrival times* of privately used vehicles exclusively for work commuting.
3	*VISTA_Time_WorkplaceArDe_WorkVehicle.csv*		Workplace *arrival & departure times* of privately used vehicles exclusively for work commuting.
4	*VISTA_Time_HomeDeAr_CasualVehicle.csv*		Home *departure & arrival times* of privately used vehicles exclusively for casual commuting.
5	*VISTA_PD_HomeDeAr _WorkVehicle.csv*	Processed datasets	The probability distributions of the home *departure & arrival times* for work commuting vehicles.
6	*VISTA_PD_WorkplaceArDe _WorkVehicle.csv*		The probability distributions of the workplace *arrival & departure times* for work commuting vehicles.
7	*VISTA_PD_HomeDeAr_CasualVehicle.csv*		The probability distributions of the home *departure & arrival times* for casual commuting vehicles.

### 3.1 T_VISTA1218_V1.csv (including VISTA - Glossary of Variables 12–18.docx)

The raw dataset is the travel survey data contained in the file *T_VISTA1218_V1.csv*. Detailed information about this has been introduced in [6].

### 3.2 VISTA_Time_HomeDeAr_WorkVehicle.csv

In the original research article [1], the privately-owned vehicles are divided into two categories as follows:

• *Work vehicle*: these are vehicles mainly used for commuting to and from work*, and*
• *Casual vehicle*: these are vehicles mainly used for other daily activities (shopping, education, etc.).

*VISTA_Time_HomeDeAr_WorkVehicle.csv* is a filtered dataset that describes the home *departure time* and home *arrival time* of *work vehicle*s. In this filtered dataset, there are 10,855 data entries for home *departure time* and 10,505 data entries for home *arrival time*. The difference between the number of data items for home *departure times* and home *arrival times* of *work vehicle* is not known; these are the numbers obtained from the original raw dataset [2]. It is possible that some of the users forgot to record the data on some days. The difference between the number of data items for *departure* and *arrival times* does not pose a problem, because the two probability distributions created for *departure* and *arrival times* are independent of each other. Table 2 provides the description of each column in the dataset.

**Table 2:** Description of dataset: *VISTA_Time_HomeDeAr_WorkVehicle.csv*.

Column #	Name	Description (in minutes, starting from midnight.)
1	DEPTIME	Time of departure from home for *work vehicles*
2	ARRTIME	Time of arrival at home for *work vehicles*

### 3.3 VISTA_Time_WorkplaceArDe_WorkVehicle.csv

*VISTA_Time_WorkplaceArDe_WorkVehicle.csv* is a filtered dataset that captures the workplace *arrival time* and workplace *departure time* of *work vehicle*s. In this filtered dataset, there are 15,512 data entries for workplace *departure time* and 15,730 data entries for workplace *arrival time*. Table 3 provides the description of each column in the dataset.

**Table 3:** Description of dataset: *VISTA_Time_WorkplaceArDe_WorkVehicle.csv*.

Column #	Name	Description (in minutes, starting from midnight.)
1	ARRTIME	Time of arrival at workplace for *work vehicles*
2	DEPTIME	Time of departure from workplace for *work vehicles*

### 3.4 VISTA_Time_HomeDeAr_CasualVehicle.csv

*VISTA_Time_HomeDeAr_CasualVehicle.csv* is a filtered and categorised dataset that describes the home *departure time* and home *arrival time* of *casual vehicle*s. Considering the different daily travel frequencies of the casual vehicles in this dataset, the vehicles are divided into the following three categories:

• *Once-a-day travel*: 76 % of *casual vehicles* depart from home only once and arrive at home after completing all trips for the day,
• *Twice-a-day travel*: 19 % of *casual vehicles* depart from and arrive at home twice each day,
• *Thrice-a-day travel*: 5 % of *casual vehicles* depart from and arrive at home three or more times a day.

**Fig. 1:**
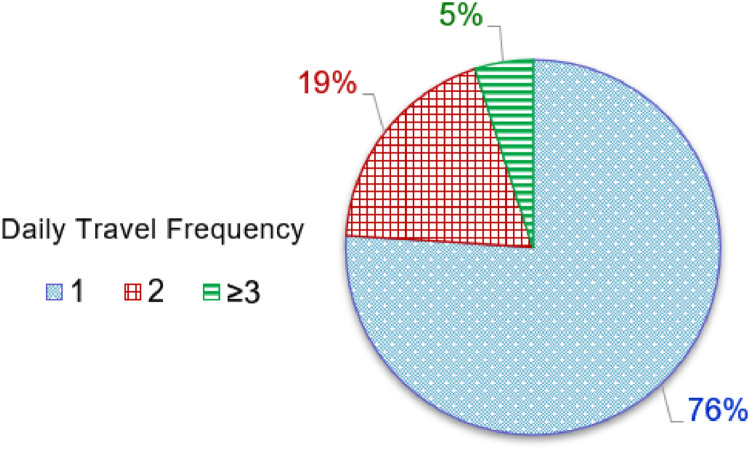
Daily travel frequencies of *casual vehicles*.

Fig. 1 shows the proportions of the daily travel frequencies. In this dataset, for *once-a-day travel*, there are 11,811 data entries for home *departure time* and 12,482 data entries for home *arrival time*; for *twice-a-day travel*, there are 2940 data entries for each of the home *departure times* and 3100 data entries for each of the home *arrival times*; and for *thrice-a-day travel*, there are 790 data entries for each of the home *departure times* and 818 data entries for each of the home *arrival times*. In calculating the percentages shown in Fig. 1, the average of the numbers of home *departure* and *arrival times* in each category were used. Table 4 provides the description of each column in the dataset.

**Table 4:** De*scription of dataset: VISTA_Time_HomeDeAr_CasualVehicle.csv*.

Column #	Name	Description (in minutes, starting from midnight.)
1	Once_DEPTIME	Time of departure from home for *casual vehicles* with once-a-day travel
2	Once_ARRTIME	Time of arrival at home for *casual vehicles with* once-a-day travel
3	Twice_1st_DEPTIME	Time of 1^st^ departure from home for *casual vehicles* with twice-a-day travel
4	Twice_2nd_DEPTIME	Time of 2^nd^ departure from home for *casual vehicles with* twice-a-day travel
5	Twice_1st_ARRTIME	Time of 1^st^ arrival at home for *casual vehicles with* twice-a-day travel
6	Twice_2nd_ARRTIME	Time of 2^nd^ arrival at home for *casual vehicles with* twice-a-day travel
7	Thrice_1st_DEPTIME	Time of 1^st^ departure from home for *cas*ual vehicles with thrice-a-day *travel*
8	Thrice_2nd_DEPTIME	Time of 2^nd^ departure from home for *cas*ual vehicles with thrice-a-day *travel*
9	Thrice_3rd_DEPTIME	Time of 3^rd^ departure from home for *cas*ual vehicles with thrice-a-day *travel*
10	Thrice_1st_ARRTIME	Time of 1^st^ arrival at home for *cas*ual vehicles with thrice-a-day *travel*
11	Thrice_2nd_ARRTIME	Time of 2^nd^ arrival at home for *cas*ual vehicles with thrice-a-day *travel*
12	Thrice_3rd_ARRTIME	Time of 3^rd^ arrival at home for *cas*ual vehicles with thrice-a-day *travel*

### 3.5 VISTA_PD_HomeDeAr_WorkVehicle.csv

**Fig. 2:**
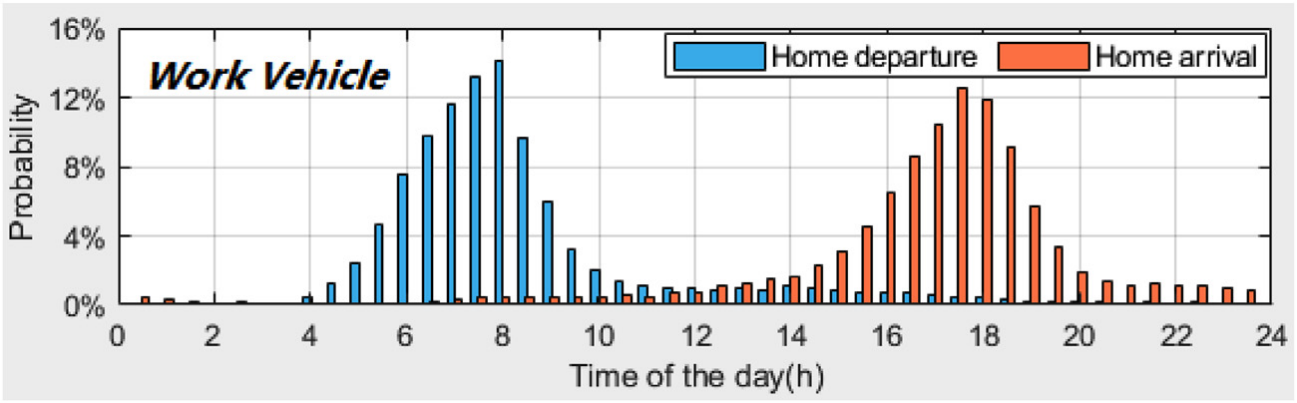
Probability distributions of home *departure times* and home *arrival times* of *work vehicles*.

*VISTA_PD_HomeDeAr_WorkVehicle.csv* contains the probability distributions of the home *departure times* and home *arrival times* of *work vehicles*. Fig. 2 illustrates these two distributions. A time interval of 30 min is used to calculate the probability distributions. Table 5 provides the description of each column in the dataset.

**Table 5:** Description of dataset: *VISTA_PD_HomeDeAr_WorkVehicle.csv*.

Column #	Name	Description
1	INDEX_TIME	Index of time (30-minute time interval, 48 time plots starting from midnight)
2	PD_DEPTIME	Probability distribution of home *departure times* for *work vehicles*
3	PD_ARRTIME	Probability distribution of home *arrival times* for *work vehicles*

### 3.6 VISTA_PD_WorkplaceArDe_WorkVehicle.csv

**Fig. 3:**
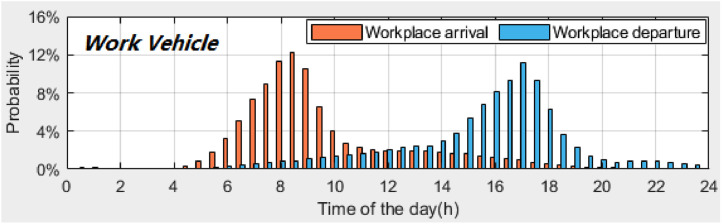
Probability distributions of workplace *arrival times* and workplace *departure times* of *work vehicles*.

*VISTA_PD_WorkplaceDeAr_WorkVehicle.csv* contains the probability distributions of the workplace *arrival times* and workplace *departure times* of *work vehicles.* Fig. 3 illustrates these two distributions. A time interval of 30 min is used to calculate the probability distributions. Table 6 provides the description of each column in the dataset.

**Table 6:** Description of dataset: *VISTA_PD_WorkplaceArDe_WorkVehicle.csv*.

Column #	Name	Description
1	INDEX_TIME	Index of time (30-minute time interval, 48 time plots starting from midnight)
2	PD_ARRTIME	Probability distribution of workplace *arrival times* for *work vehicles*
3	PD_DEPTIME	Probability distribution of workplace *departure times* for *work vehicles*

### 3.7 VISTA_PD_HomeDeAr_CasualVehicle.csv

**Fig. 4:**
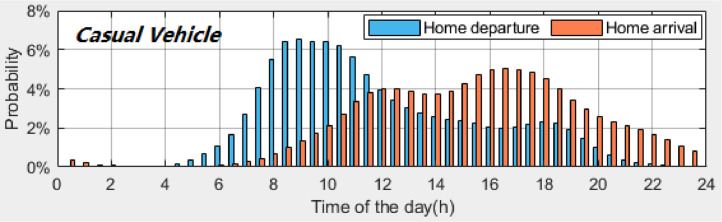
Probability distributions of home *departure times* and home *arrival times* of *casual vehicles* with *once-a-day travel*.

**Fig. 5:**
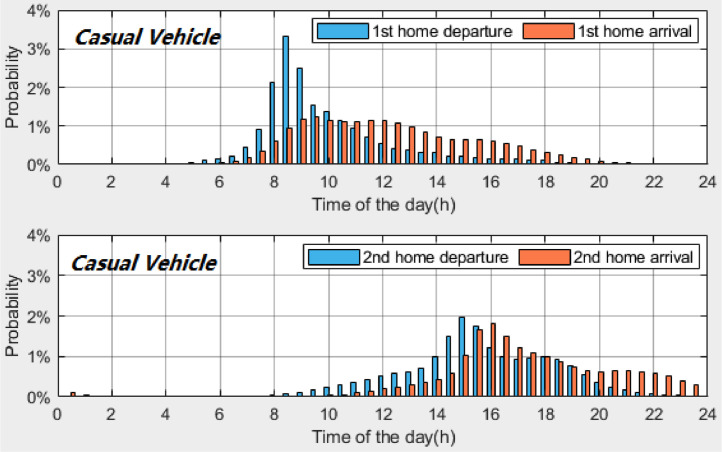
Probability distributions of home *departure times* and home *arrival times* of *casual vehicles* with *twice-a-day travel*.

**Fig. 6:**
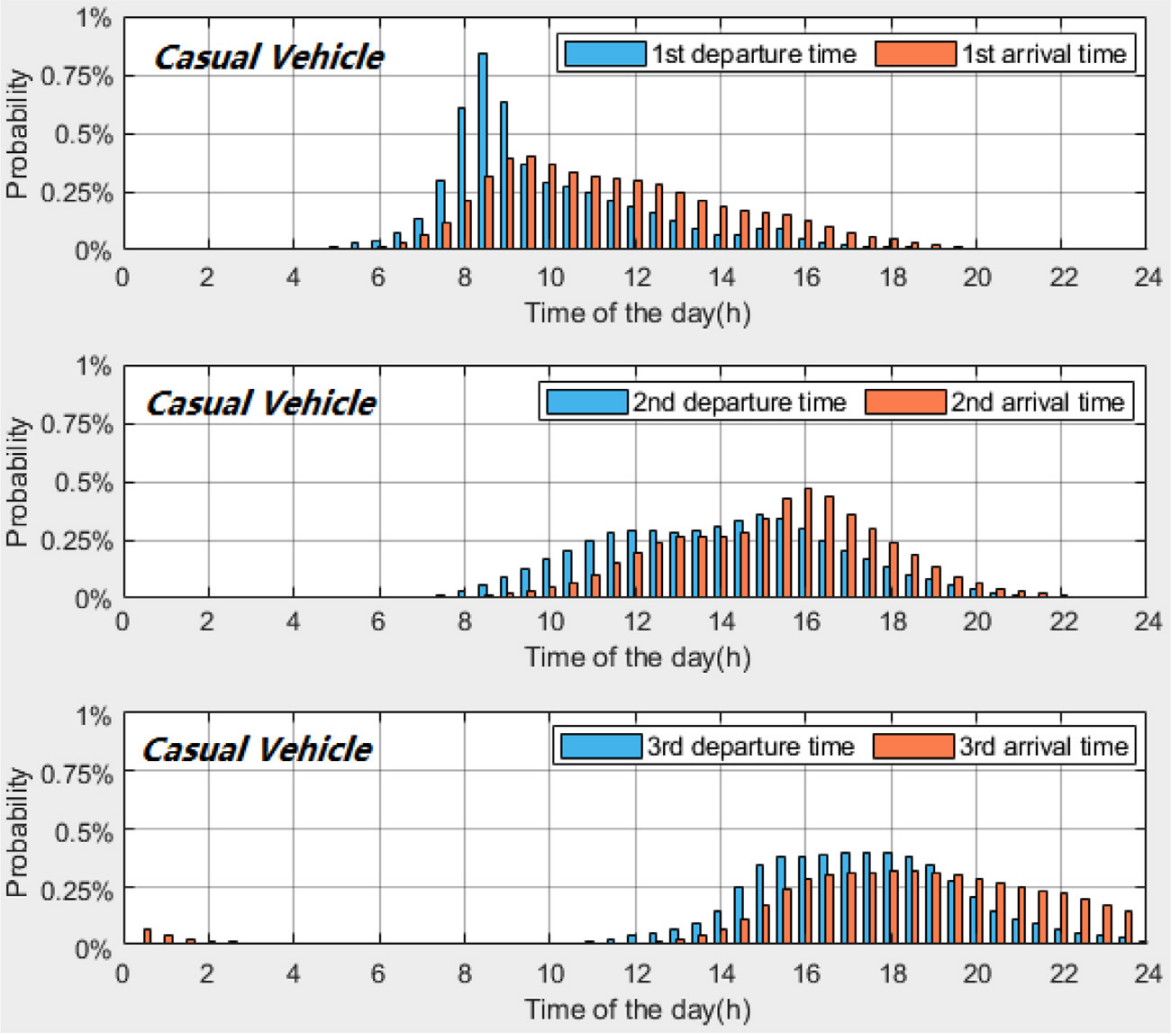
Probability distributions of home *departure times* and home *arrival times* of *casual vehicles* with *thrice-a-day travel*.

*VISTA_PD_HomeDeAr_CasualVehicle.csv* contains the probability distributions of the home *departure* and *arrival times* of *casual vehicles*. Table 7 provides the description of each column in the datasets. Fig. 4, Fig. 5, Fig. 6 present the probability distributions of *casual vehicles* with *once-a-day travel, twice-a-day travel* and *thrice-a-day travel*, respectively. A time interval of 30 min is used to calculate the probability distributions.

**Table 7:** Description of dataset: *VISTA_PD_HomeDeAr_CasualVehicle.csv*.

Column #	Name	Description
1	INDEX_TIME	Index of time (30-minute time interval, 48 time plots starting from midnight)
2	PD_Once_DEPTIME	Probability distribution of home *departure times* for *casual vehicles* with once-a-day travel
3	PD_Once_ARRTIME	Probability distribution of home *arrival times* for *casual vehicles* with once-a-day travel
4	PD_Twice_1st_DEPTIME	Probability distribution of 1^st^ home *departure times* for *casual vehicles* with twice-a-day travel
5	PD_Twice_2nd_DEPTIME	Probability distribution of 2^nd^ home *departure times* for *casual vehicles* with twice-a-day travel
6	PD_Twice_1st_ARRTIME	Probability distribution of 1^st^ home *arrival times* for *casual vehicles* with twice-a-day travel
7	PD_Twice_2nd_ARRTIME	Probability distribution of 2^nd^ home *arrival times* for *casual vehicles* with twice-a-day travel
8	PD_Thrice_1st_DEPTIME	Probability distribution of 1^st^ home *departure times* for *casual vehicles* with *thrice-a-day travel*
9	PD_Thrice_2nd_DEPTIME	Probability distribution of 2^nd^ home *departure times* for *casual vehicles* with *thrice-a-day travel*
10	PD_Thrice_3rd_DEPTIME	Probability distribution of 3^rd^ home *departure times* for *casual vehicles* with *thrice-a-day travel*
11	PD_Thrice_1st_ARRTIME	Probability distribution of 1^st^ home *arrival times* for *casual vehicles* with *thrice-a-day travel*
12	PD_Thrice_2nd_ARRTIME	Probability distribution of 2^nd^ home *arrival times* for *casual vehicles* with *thrice-a-day travel*
13	PD_Thrice_3rd_ARRTIME	Probability distribution of 3^rd^ home *arrival times* for *casual vehicles* with *thrice-a-day travel*

## 4 Experimental Design, Materials and Methods

This paper only presents travel datasets used in the research article [1]. In addition to the travel datasets the research article also used household load profile and PV generation data which are not discussed in this paper, but similar load profile and PV generation data can be found in [13].

The methods used to process the datasets are implemented using the Microsoft Excel and MATLAB software. The experimental method for each dataset is described below.

### 4.1 Select and download raw data

The raw travel dataset *T_VISTA1218_V1.csv* and the associated instruction file *VISTA - Glossary of Variables 12–18.docx* are downloaded from the Victorian Integrated Survey of Travel & Activity [2]*.* The method used to select and download the travel survey data was introduced in [6]. In the raw dataset (*T_VISTA1218_V1.csv*), every trip is assigned a Trip ID, which identifies the trip by specifying the year, house ID, ID of the person making the trip (person ID) and the trip number. Examples and corresponding descriptions have been presented in [6].

### 4.2 Filter data for departure and arrival times

Excel's built-in *filtering* function and the MATLAB software are used on the raw dataset to obtain the datasets for *departure* and *arrival times* for both *work vehicles* and *casual vehicles*.

#### 4.2.1 Work vehicles

**Fig. 7:**
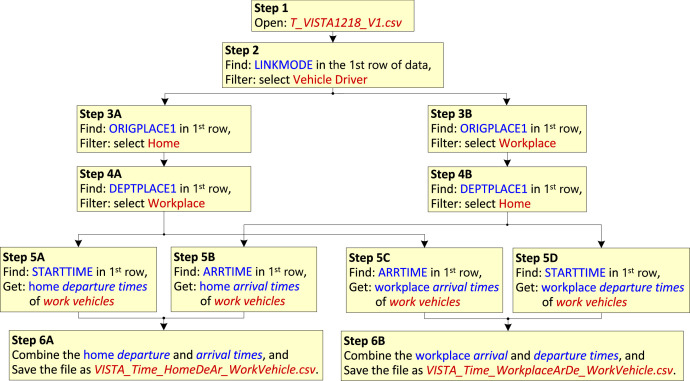
Processing steps to obtain *departure* and *arrival times* of Work Vehicles for the datasets *VISTA_Time_HomeDeAr_WorkVehicle.csv* and *VISTA_Time_WorkplaceArDe_WorkVehicle.csv*.

For *work vehicles*, Fig. 7 shows the steps used to obtain the datasets described in 4.2, 4.3, namely, *VISTA_Time_HomeDeAr_WorkVehicle.csv* and *VISTA_Time_WorkplaceArDe_WorkVehicle.csv*.

The processing accomplished in the steps shown in Fig. 7 are summarised next:

**Step 1–2:** Open the raw data file *T_VISTA1218_V1.csv*. Find LINKMODE in the first row, use the *filter* function to select ‘Vehicle Driver’. When ‘Vehicle Driver’ is selected then the travel data belonging only to the drivers of privately used vehicles are retained and the data for other types of commuting modes (such as bus, walking, passenger of private vehicle, and train) are excluded.
**Step 3–4:** Find ORIGPLACE1 in the first row of the dataset and use the *filter* function to select Home/Workplace. Then find DEPTPLACE1 in the first row of the dataset and use the *filter* function to select Workplace/Home. This will allow the selection of the origin and destination of vehicles commuting between the home and the workplace.
**Step 5:** The home *departure* and *arrival times* of the *work vehicles* are obtained from the columns STARTTIME and ARRTIME of the dataset as shown by Steps 5A and 5B respectively. The workplace *arrival* and *departure times* of the *work vehicles* are obtained from the columns ARRTIME and STARTTIME as shown by Steps 5C and 5D respectively.
**Step 6:** Use Step 6A to combine the home *departure times* and *home arrival times* obtained in Steps 5A and 5B into one dataset and save the file as *VISTA_Time_HomeDeAr_WorkVehicle.csv*. Use Step 6B to combine the workplace *arrival times* and workplace *departure times* obtained in Steps 5C and 5D into one dataset and save the file as *VISTA_Time_WorkplaceArDe_WorkVehicle.csv*.

#### 4.2.2 Casual vehicles

**Fig. 8:**
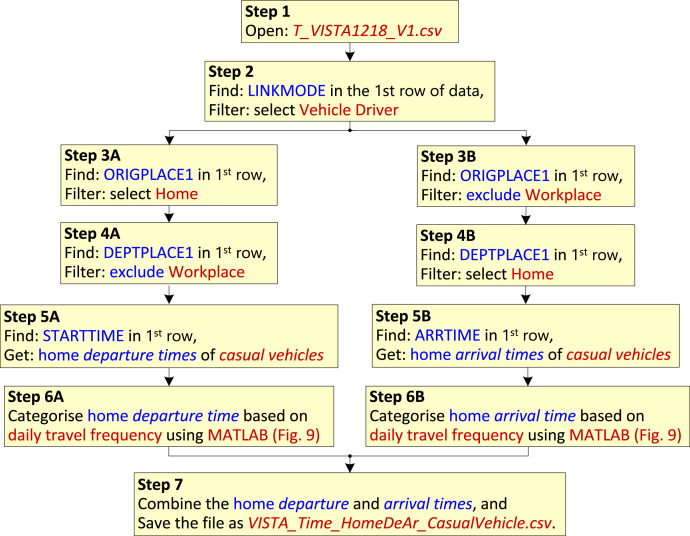
Flowchart of processing to obtain the dataset *VISTA_Time_HomeDeAr_CasualVehicle.csv*.

**Fig. 9:**
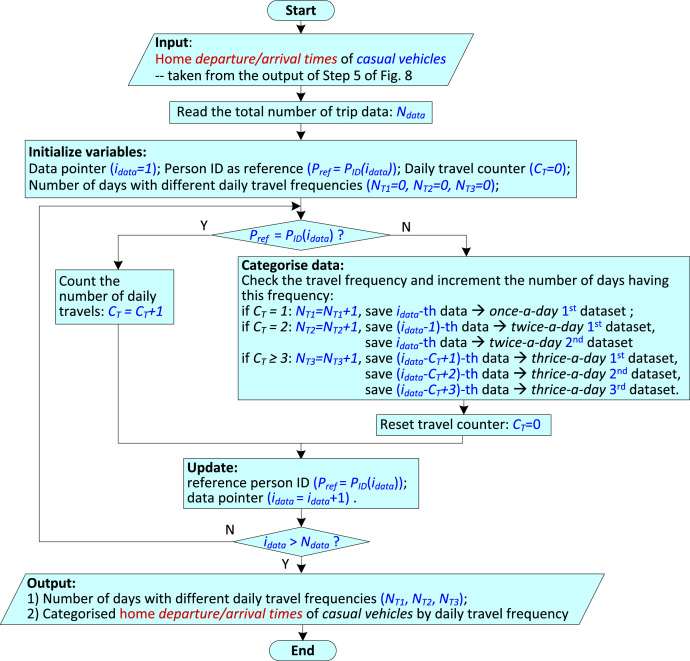
Flowchart of MATLAB data processing program for categorising the *casual vehicle* dataset.

For *casual vehicles*, Fig. 8 shows the steps used to obtain the dataset described in Section 3.4, namely, *VISTA_Time_HomeDeAr_CasualVehicle.csv*. As per the original research article [1], *casual vehicles* can have variable daily travel frequencies. In Step 6 of Fig. 8, a MATLAB data processing program is used to divide the home *departure/arrival times* into three categories based on the daily travel frequency. The flowchart of that program is presented in Fig. 9.

In Fig. 8, the first two steps are the same as those in Fig. 7, to select data related to the drivers of privately used vehicles only. Steps 3 and 4 are used to select data related to only the *casual vehicles*; and, upon finding ORIGPLACE1/DEPTPLACE1 in the first row of the dataset, use the *filter* function to select Home or to exclude Workplace. The home *departure* and *arrival times* of the *casual vehicles* are obtained from the columns STARTTIME and ARRTIME of the dataset as shown by Steps 5A and 5B respectively. Then, Step 6 uses the MATLAB data categorization program of Fig. 9 as mentioned in the previous paragraph. Finally, in Step 7, the categorised home *arrival times* and home *departure times* are combined into one dataset, and the file is saved as *VISTA_Time_HomeDeAr_CasualVehicle.csv*.

Fig. 9 presents the flowchart of the MATLAB data processing program for categorising the *casual vehicles* based on daily travel frequency. The various processing steps are described next:

• The home *departure/arrival times* of the *casual vehicles* obtained from Step 5 of Fig. 8 are used as the input.
• A check is performed to see if the *person ID* for two adjacent home *departure/arrival times* are the same; if the adjacent times belong to the same person, *P_ref_=P_ID_* (*i_data_*), then the daily travel counter (*C_T_*) is incremented; however, if they don't belong to the same person then the accumulated daily travel counter is used to determine the daily travel frequency. The number of days having this travel frequency (*N_T1_, N*_T2_ or *N_T3_*) is incremented, and the home *departure/arrival times* are saved to the corresponding dataset.
• Finally, two outputs are obtained: 1) the number of days with different daily travel frequencies (*N_T1_, N*_T2_, *N_T3_*), and 2) categorised home *departure/arrival times* of *casual vehicles* by daily travel frequency. The first output is used to calculate the proportion of each daily travel frequency, as shown in Fig. 1, and the second output is used to obtain the dataset *VISTA_Time_HomeArDe_CasualVehicle.csv*.

### 4.3 Extract probability distribution

For *departure* and *arrival times* obtained in Section 4.2, the corresponding probability distributions are calculated using the MATLAB built-in function *‘ksdensity’*, and saved in *VISTA_PD_HomeDeAr_WorkVehicle.csv, VISTA_PD_WorkplaceArDe_WorkVehicle.csv* and *VISTA_PD_HomeDeAr_CasualVehicle.csv* respectively. In the original study, the time interval is set to 30 min; the amount of time interval can be chosen according to requirements.

## Limitations

One limitation of the dataset is that the original dataset obtained from VISTA [2] does not provide any date-related information, making it impossible to distinguish between the driving demands on workdays and non-workdays. The second limitation is that the raw dataset (*T_VISTA1218_V1.csv*) does not provide any vehicle ID. Therefore, if the same vehicle commutes between home and different destinations in different trips then these trips can be potentially regarded as trips by different vehicles. Nonetheless, because it is possible to filter the data based on travel category, i.e. *work* or *casual*, and obtain separate dataset for each, therefore the obtained probability distributions of *departure* and *arrival times* can be assumed to be representative of the travel patterns of the corresponding vehicle categories and travel destinations.

## Ethics Statement

The authors have read and followed the ethical requirements for publication in *Data in Brief* and confirm that the current work does not involve human subjects, animal experiments, or any data collected from social media platforms. The primary data sourced from the Victorian Integrated Survey of Travel & Activity (VISTA) is openly accessible through the VISTA website [2]. As such, the authors did not need permission to use the data.

## CRediT Author Statement

**Yan Wu:** Conceptualization, Methodology, Investigation, Software, Data curation, Visualization, Writing - original draft, Writing - review & editing. **Syed Mahfuzul Aziz:** Supervision, Conceptualization, Methodology, Visualization, Project administration, Writing - review & editing. **Mohammed H. Haque:** Supervision, Conceptualization, Methodology, Visualization, Writing - review & editing.

## Data Availability

Data for: Travel datasets to analyse the impacts of Vehicle-to-Home operation and multi-location charging of electric vehicles on household energy cost (Original data) (Mendeley Data).
